# What Causes Health Information Avoidance Behavior under Normalized COVID-19 Pandemic? A Research from Fuzzy Set Qualitative Comparative Analysis

**DOI:** 10.3390/healthcare10081381

**Published:** 2022-07-25

**Authors:** Qingxiu Ding, Yadi Gu, Gongrang Zhang, Xingguo Li, Qin Zhao, Dongxiao Gu, Xuejie Yang, Xiaoyu Wang

**Affiliations:** 1 School of Management, Hefei University of Technology, Hefei 230009, China; ac14dqx@163.com (Q.D.); grzhang118@126.com (G.Z.); lixingguo@hfut.edu.cn (X.L.); xuejie_y@126.com (X.Y.); 2 Mental Health Education and Counseling Center, University of Shanghai for Science and Technology, Shanghai 200093, China; yadigu2002@163.com; 3 School of Foreign Studies, Hefei University of Technology, Hefei 230009, China; feliciazhao@hfut.edu.cn; 4 The Department of Pharmacy of the 1st Affiliated Hospital, Anhui University of Chinese Medicine, Hefei 230031, China

**Keywords:** health information, information avoidance behavior, fuzzy-set qualitative comparative analysis, normalized COVID-19 pandemic

## Abstract

Affected by the normalization of the COVID-19 pandemic, people’s lives are subject to many restrictions, and they are under enormous psychological and physical pressure. In this situation, health information may be a burden and cause of anxiety for people; thus, the refusal of health information occurs frequently. Health-information-avoidance behavior has produced potential impacts and harms on people’s lives. Based on more than 120,000 words of textual data obtained from semi-structured interviews, summarizing a case collection of 55 events, this paper explores the factors and how they combine to lead to avoidance of health information. First, the influencing factors are constructed according to the existing research, and then the fuzzy set qualitative comparative analysis (fsQCA) method is used to discover the configuration relationship of health-information-avoidance behavior. The results show that the occurrence of health-information avoidance is not the result of a single factor but the result of a configuration of health-information literacy, negative emotions, perceived information, health-information presentation, cross-platform distribution, and the network information environment. These findings provide inspiration for reducing the adverse consequences of avoiding health information and improving the construction of health-information service systems.

## 1. Introduction

Health is an important topic that is closely related to everyone, which has attracted growing attention in China, as it is an indispensable connotation of building a modern socialist country in an all-round way for the pursuit of sustainable development and happiness of human society. Health information can regulate people’s health beliefs, prompt people to form healthy behavior concepts, and guide them to maintain, enhance, or change unhealthy behaviors [1]. Therefore, health-information-seeking behavior is usually used to reveal users’ information need. However, as Maslow said, we can reduce anxiety by seeking knowledge, and we can also reduce anxiety by avoiding knowing [2]. People will defend their attitudes, beliefs, and behaviors by avoiding information that challenges them and seeking information that supports them, thereby, eliminating or reducing cognitive dissonance [3]. 

Since the outbreak of the COVID-19 pandemic, facing a long period of normalized epidemic prevention and control, people in many medium- and high-risk areas have to stay at home or have had restricted travel imposed. These long-term closures and control measures have put many people under enormous physical and psychological dual pressure. Health information about the development of the epidemic, the number of confirmed cases, disease manifestations, sequelae, etc. have gone viral on the internet. While people can easily obtain this information, they are worried at the same time. 

People’s attitudes to health information are not all positive, and negative avoidance also occurs frequently. Chen et al. focused on nurses from low-risk areas and that the mental health status outcomes were poor [4]. During a global health crisis, the health information behavior is from information seeking to information avoidance [5]. Researchers have shown that the double burden of a COVID-19 knowledge deficit is low health literacy and high information avoidance [6]. In a multicountry comparative study, misinformation exposure reduced information insufficiency, which subsequently led to greater information avoidance [7]. 

Health-information-avoidance behavior is receiving increased attention from researchers. Sairance and Savolainen conducted a semi-structured interview with university students and found that health information was primarily avoided because of the risk of experiencing negative emotions, such as fear, anxiety, and depression, or to face information that is unsuitable for one’s needs based on uncertainty management as a framework [8]. Howell et al. found that people who were short of personal and interpersonal resources were more likely to avoid learning potentially-threatening information when dealing with threat [9]. 

Taber et al. concluded that information avoidance tendencies might influence decisions to receive threatening health information; threat management resources hold promise for mitigating this association [10]. These theories are often applied to analyze the problem of information avoidance. In health context, health information avoidance encompasses a variety of actions, including avoiding visiting the doctor [11], screening [12,13], learning risk for cancer information [13], and information about one’s prognosis [14]. 

This behavior represents a defensive response [15]; however, such avoidance also can be problematic. For instance, delaying diagnosis can have dire health consequences for diseases, such as cancer or HIV, for which early initiation of treatment can substantially prolong life [16]. Howell and Shepperd examined whether social exclusion might prompt health information avoidance as the painful experience of rejection could lead to people to make ego-defensive decisions, such as avoiding information [17]. 

Although some scholars have identified the factors influencing information avoidance (for instance, information overload [18], perceived control, and convenience of obtaining or interpreting information [19]), few studies have examined the effects of different combinations of factors on health-information-avoidance behavior. 

**Figure 1:**
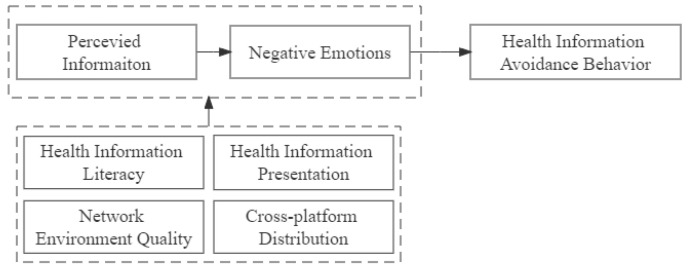
Research variables.

Therefore, this paper aims to explore the key factors that lead to health-information avoidance and to discuss the effects of different combinations of these factors. Specifically, we select relevant conditions and determine how they are combined to influence the behavior (see Figure 1) using fsQCA based on true health information avoidance experiences. This study makes two key contributions. First, it shows that the occurrence of health information avoidance behavior is the result of a combination of internal and external factors. 

Individual cognition and negative emotions as internal factors will be affected by personal literacy, which is also an internal factor, while the presentation forms and characteristics of health information and the network information environment are external factors that act together. Second, different from the linear relationship and net effects of causality in the traditional regression analysis method, this paper uses the qualitative comparative analysis to fully explore the causal complexity of health information avoidance. 

The structure of this paper is organized as follows. The Section 2 presents the literature review, and the Section 3 describes the research method, variables derived from prior discussions and data collected. The Section 4 introduces the analysis results, and discussions are described in Section 5. The Section 6 includes our conclusions, implications, limitations, and suggestions for future research. 

## 2. Literature Review

### 2.1. Definition of Health-Information-Avoidance Behavior

Health information behavior is a derived concept of information behavior. Information behavior studies hold that people strive to find information to acquire knowledge, reduce uncertainty, and make decisions that guide behavior [20,21]. Health information behavior refers to the sum of various information behaviors of people using health information in a certain situation, which is becoming a research hotspot in this field at present [22,23]. However, an individual’s awareness of the need for health information governs the behavior, which may be search, use, evaluation, or avoidance [24]. 

Information avoidance is “any action designed to prevent or delay access to information that is available but may not be desired” [19]. Health-information-avoidance behavior is a subcategory of health information behavior. The health-information-avoidance behavior discussed in this article refers to health information, which is affected by a variety of internal and external factors in the context of health information, taking health information as the object of avoidance or actively or passively occurring in any period of the life cycle of health information. 

### 2.2. Drives and Tactics of Health-Information-Avoidance Behavior

Avoiding health information often occurs in medical and health situations. In Finland, using multiple sources to obtain information and information overload resulted in information avoidance during the global health crisis (COVID-19) [5]. In China, anxiety and cognitive dissonance related to COVID-19 increased consumers’ information avoidance intension [18]. Avoidance of cancer information is also typical. Usually, people tended to avoid genetic screening information that pointed to cancer [25]. Further research showed that older, less educated, lower household income, unemployed, disabled or retired, and uninsured people were unwilling to know cancer information in the hopes of reducing anxiety and maintaining hope [26]. 

Coping with information overload and reducing cognitive load are considered common motivations for information avoidance. Some studies on information systems have found that information avoidance is an effective means of dealing with information overload [27,28]. When Twitter users are challenged with information overload, they prevent seeing all the tweets they receive, instead of reducing their friend count to reduce the total number of tweets they receive [29]. 

The information overload perceived by users in public health emergencies produces negative emotions in the users and exaggerates the possibility of their own infection with the novel coronavirus, which will lead to users’ defensive psychology against relevant information and reduce contact behavior with information [30]. Tactics are the measures to achieve health-information avoidance. The direct avoidance of information sources, control of attention, delayed access to information, and forgetting and denial of information are common tactics [31]. Furthermore, selective avoidance [32,33], passive avoidance [19,34,35], and temporary avoidance [36,37] are also information avoidance types. In addition, language avoidance is also a form of concealment in verbal or written communication. Faced with the cancer health information questionnaire, many people prefer the response of “I don’t know” [38,39]. 

### 2.3. Explanation of the Factors Affecting Health Information Avoidance Behavior

Previous studies have shown that the influencing factors of health-information-avoidance behavior mainly include individual factors, information factors, and situational factors. Individual factors refer to factors related to the physiology, psychology, ability, and personality of the behavior subject, such as personal characteristics, cognitive conflicts [40], perceived control [41], and coping resources [42]. Basic demographic characteristics, such as gender, age, education level, and health status, are basic variables that are commonly used in statistical research on the distribution of health information avoidance behavior characteristics. 

For example, the wiliness of the elderly to avoid health information is more obvious [35,43], less educated and low income groups are more likely to avoid health information [44,45], trait anxiety is a strong predictor of cancer information avoidance [46], and people with Type II diabetes tend to avoid health information due to psychological stress [47]. Studies with information systems have shown that the emotional aspects of group effectiveness can play a moderating role, suggesting that hospital management pay more attention to the potential negative impact of emotional mitigation on the organization [48].

Health information literacy includes health awareness, knowledge, confidence, and information literacy, which directly or indirectly affects individuals’ behavior performance by their cognition and emotions on self-efficacy, perceived usefulness, perceived availability, perceived risks, and negative outcomes. Individuals with lower health-information literacy are more likely to have health information anxiety due to the inability to understand and judge true or false information, and thus they avoid health information [49]. Social media users’ perceived information overload creates exhaustion, which in turn promotes information avoidance intentions [50]. Information irrelevance directly led to information avoidance behavior, and negative effects played a mediating role [51]. On the contrary, guided by the risk information-seeking and processing model, information avoidance appeared to be driven by positive effects [52]. 

Information factors refer to the characteristics of information itself that affect health-information-avoidance behavior as an external objective factor. Information overload and information quality are frequently mentioned variables. Information overload means that “situations in which we receive too much information to sensibly deal with it all in our available time frame” [53]. Excessive information may burden people instead of acquiring more accurate intelligence or knowledge. 

Individuals need to spend a great deal of energy and resources to process and analyze health information, which can easily lead to information fatigue, resulting in negative emotions and experiences particularly if the information is mis/dis-information or health rumors, which can be dangerous as this can lead to a critical situation [54]. However, the internet has become an ideal breeding ground for the spread of fake news, misleading information, fake reviews, rumors, etc. [55]. For example, in the US, South Korea, and Singapore, exposure to misinformation about COVID-19 reduced insufficient information but then led to greater information avoidance [7]. 

As health misinformation is more closely related to health issues that people care about in public health events, such as the conspiracy theories, hoaxes, and falsehoods regarding COVID-19 going viral on social media, it has a greater impact on negative emotions, such as health anxiety and avoidance [56]. Information quality includes many dimensions, such as reliability, objectivity, usefulness, completeness, and relevance [57]. If the perceived quality is too poor or impossible to judge, cognitive conflicts and pressure affect the intention of continuing to use online health information. 

At the same time, the perceived quality of health information affects the perception of one’s own potential health risks. Exaggerated illness and inconsistent health information can easily lead to health anxiety, resulting in avoiding health information to reduce anxiety. Message framing is a form of information expression that influences decision-making. In the past, most of the focus was on the impact of health information content on individual health behaviors, while the relationship between health information expression and health behaviors has not received sufficient attention [58]. Thus, the health message framing is worth examining. 

In addition, with the development of mobile internet, the role of information sources in health-information avoidance has been given increasing attention. With the rise of the mobile internet, medical health applications, such as Chunyu Doctor, Baidu, and Pomelo, are increasingly used by the general public. As a result, mobile health and mobile medical services have become hotspots among scholars worldwide [59]. Websites and social media were the most frequently avoided sources of information in a study of predictors of information-avoidance behavior of German news consumers during the COVID-19 pandemic [60]. 

This was also supported by findings in Finland, where social media and personal networks were not the preferred sources of information [5]. Exposure to different types of information sources is positively associated with an individual’s sense of information overload and information anxiety, as evidenced by previous studies [61,62,63,64]. People who use social media as a source of COVID-19 information tend to feel more information overload, leading to information avoidance [65]. In China, Baidu is the largest source of health information for Chinese netizens, and WeChat is another major source of health information [66]. 

Situational factors refer to the physical environment, social environment, or a specific situation. The physical environment includes the network environment, interface friendliness, mobile devices, etc. The complex health information display interface or sudden freezes or black screens during use will cause the elderly to panic and be overwhelmed and anxious, thereby, reducing the use or directly quitting the use [67]. 

Apart from traditional search engines, social media has become a prominent information acquisition channel. A variety of health information sources make cross-platform distribution of health information a prominent feature. However, social media has become an ideal breeding ground for fake news, misleading information, fake reviews, rumors, etc., as most of its content is created by users, and not all information is released by professional doctors and medical authorities [55]. This health information environment should be discussed.

Existing studies have explored the influencing factors of health-information-avoidance behavior from different perspectives, mainly emphasizing the effect of a single factor on the avoidance results. However, there are contradictions in the influence of various factors on the results, and the source of the contradiction may be that the combined effect of other factors is ignored. Whether the behavior occurs or not is a complex and comprehensive decision-making process, and the way in which these multiple factors combine and the mechanisms by which they take place have been seldom addressed. Therefore, it is necessary to explore the configuration relationship and joint-action mechanisms among the three influencing factors related to health-information-avoidance behavior: individual, information, and environment.

## 3. Methods

### 3.1. Qualitative Comparative Analysis

Different from traditional empirical research, which focuses on the influence of a certain variable on the outcome variable, this study aims to demonstrate whether different combinations of multiple variables have the same effect on the results. Qualitative comparative analysis (QCA) is a research method suitable for establishing connections between combinations of causal conditions.

Qualitative comparative analysis conceptualizes a case as a whole composed of causal conditions, based on holism [68], which focuses on complex causal relationships between conditional configurations and outcomes [69]. It uses Boolean algebra principles and set theory to explore the effect of multiple variable combinations on results and is suitable for discussing complex linear relationships between multiple factors. According to the classification of variable characteristics, QCA includes three types: crisp-set QCA (csQCA), muti-value QCA (mvQCA), and fuzzy-set QCA (fsQCA). csQCA uses 0 or 1 to classify variables, and mvQCA is an extension to better represent classification using multi-value variables. fsQCA uses a value between 0 and 1 to represent the degree to which the variable belongs to the set—that is, the degree of subordination of variables [70]. 

QCA has been increasingly used in the field of management. Based on the fsQCA analysis of 31 provincial government portals in China, the researchers found that four groups of methods can improve websites construction performance [71]. Taking the original innovation projects of enterprises as samples and the entrepreneurial failure experiences of entrepreneurs as samples, QCA was used to analyze the multiple reasons for the failure [72,73]. In the field of healthcare, fsQCA is used to evaluate the work performance of healthcare professionals [74] and examine the uptake or impacts of public health interventions [75]. 

In this paper, fuzzy set qualitative comparative analysis method (fsQCA) is applied to analyze how the combinations of influencing factors lead to avoidance of health information from the perspective of configuration. The reasons are as follows: first, compared with the quantitative research, fsQCA can find the configuration relations of variables, and explain more of the causal complexity. Secondly, fsQCA is suitable for medium and small sample measurement analysis, ranging from 15 to 80 [76]. This article uses the case as a sample, which belongs to the category of small samples. Thirdly, fsQCA can describe the effect of the “degree” of the presence/absence of a condition on the outcome variable, which is more precise than csQCA and mvQCA. 

**Figure 2:**
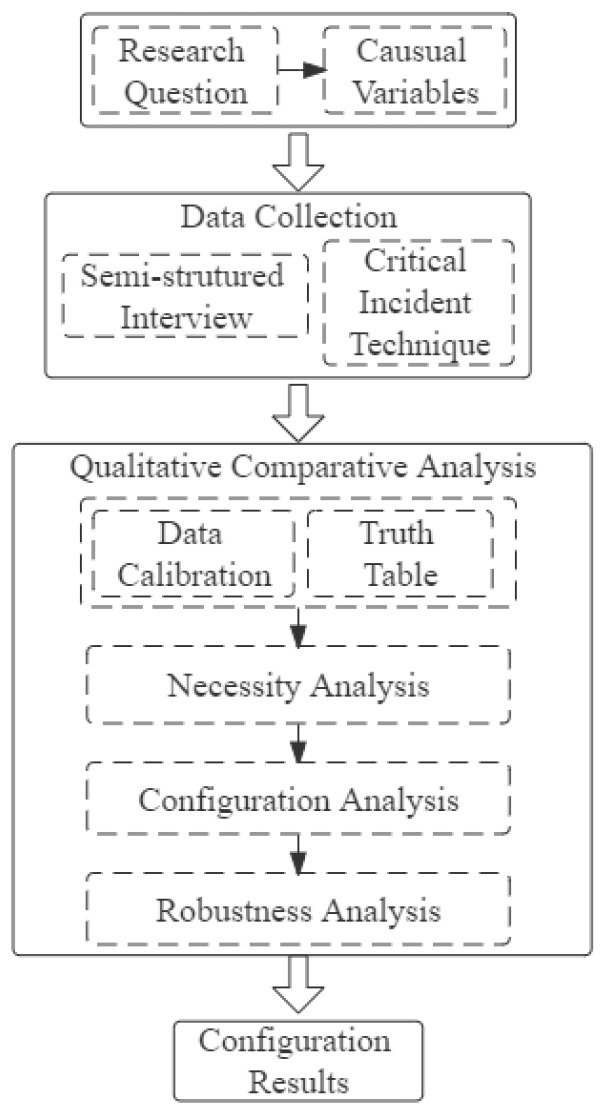
Research design flow chart.

The specific application steps of fsQCA are as follows: Define the antecedent conditions and result variables of this study. Collect data, calibrate, build the truth table, and import the fsqca3.0 software. Analyze the necessity and sufficiency of conditions and discuss the results. However, the number of antecedent conditions selected by QCA should not be too many, because the increase of condition variables will cause the exponential increase of the path, and thus it is not easy to mine the core configuration relationship. Therefore, it is better to control within the seven antecedent conditions. The overall research process was carefully designed as shown in Figure 2.

### 3.2. Sample, Cases, and Variates

The samples are the cases of avoiding health information experiences collected in semi-structured interviews using the critical incident technique (CIT). We posted recruitment posters online and recruited respondents who experienced health-information avoidance, and they described in detail the background, process, inner feelings, and emotions of an event. 

Starting from February 2021, the interviews were conducted with a combination of offline and online methods, and more than 120,000 words of textual materials and a total of 55 events obtained from interviews were used as case sets. Through offline face-to-face one-on-one interviews, it was convenient to capture the non-verbal characteristics of respondents, such as expressions, movements, etc. Affected by the epidemic, some respondents participated in the interviews through WeChat voice and video. Online interviews provided a highly personal environment that helped preserve privacy and even increase familiarity among participants.

In this process, we adhered to the highest ethical standards. Before starting the interviews, the respondents were informed about the aims, methods of data collection, and their anonymized and voluntary nature in the study. We promised to fully respect the privacy of respondents, who could refuse to talk about anything they did not want to. The study was conducted in accordance with the Declaration of Helsinki, and informed consent was obtained from the respondents.

These cases with the variable content were what we needed for the study. In order to ensure credibility, the dataset had both positive cases (in this study, avoiding health information) and negative cases (in this study, not avoiding health information). According to the literature review, six factors were selected: health-information literacy, negative emotions, perceived health information, health-information presentation, cross-platform distribution, and network environment quality. The result variable was health-information-avoidance behavior.

### 3.3. Variable Interpretation and Assignment

First, we calibrated the variables, converting to an aggregate membership score between 0 and 1. Calibration is about choosing the appropriate method based on the problem and the data. In this study, qualitative case descriptions were converted into quantitative case values. In the process, relevant theories, actual data, the knowledge of researchers, and other conditions can be used as a basis to formulate clear assignment criteria and then assign variables according to the criteria. Therefore, referring to prior studies, this paper adopts the four-value method to indirectly calibrate variables. The assignment criteria are reported in Table 1. 

#### 3.3.1. Outcome Variables

In this context, the outcome variable is health-information-avoidance behavior. Health information avoidance behavior is when an individual who faces health information, out of a certain motivation, adopts an appropriate avoidance strategy and achieves the effect of avoiding health information. This is a binary variable, where 0 means that health-information avoidance does not occur, and 1 means that health-information-avoidance behavior occurs.

#### 3.3.2. Conditional Variables

Health information literacy is important for individuals. People with health literacy and information literacy have certain health awareness and information awareness, and they can actively pay attention to health information, understand health information, and can search, find, judge, evaluate, and use relevant health information to manage their own health when needed.

Emotions can govern behaviors. As health information is likely to be some information that indicates the possibility of one’s own disease, it can easily cause negative emotional reactions in readers. Many scholars have included emotions in their research scope, such as anxiety [31], sadness [18], intension [67], horror [77], and fatigue [50].

Perceived information mainly includes health information overload, quality, and content characteristics. Additionally, the presentation of health information is also important [78]. For the same colorectal cancer screening information, the plain text presentation form is more likely to cause the elderly to avoid cancer screening than the graphical method [42]. This framing effect of health information can influence decision preferences and is worth discovering. In this paper, the message framing mainly refers to the real and clear picture format.

Cross-platform distribution is a typical characteristic of health information environment. In the current network information environment, the channels for people to obtain health information have greatly increased from the traditional sources represented by search engines that were commonly used in the past to today’s mobile social media, online health communities, professional health APPs, and other diversified Web2.0 channels. The threshold for people to obtain health information is lowering, and the amount of information obtained is increasing. 

The abundance of information sources brings about the prominent feature of cross-platform distribution of health information. On the one hand, it is convenient for people to obtain health information, and there are more possibilities in the context of information behavior. On the other hand, a variety of information sources also results in the increase of health rumors, advertisements, false health information, etc., and increases the challenges for people’s judgment and utilization of information. Thus, network environment quality refers to this health information environment where health mis/dis-information, rumors, and other bad information exist.

We assigned the case according to the assignment standard, saved it as a file, and imported it into the fsqca3.0 software for analysis.

## 4. Results

### 4.1. Single-Factor Necessity Analysis

Necessity analysis is testing whether a single condition constitutes a necessary condition for the outcome variable. A necessary condition means that the condition always occurs when the result exists—in other words, the result cannot occur without the condition. This ensures that each explanatory variable cannot explain the result alone [70]. Typically, the consistency threshold for necessary conditions defaults to 0.9 [79]—that is, the condition whose consistency exceeds 0.9 is considered as a necessary condition for the result to occur. 

We chose antecedent conditions and their non-conditions in the fsqca3.0 software and took the avoidance of health information as the result. With operation of the necessity analysis function of the software, the results are shown in Table 2. The results of the necessity analysis shows that the consistency test values of the six antecedent conditions included in the analysis and their non-conditions do not exceed the consistency threshold of 0.9, indicating that none of the 12 conditions are necessary for the occurrence of health-information-avoidance behavior. This indicates that the occurrence of health-information avoidance cannot be determined by one factor, and multiple factors should be taken into consideration.

### 4.2. Sufficiency Analysis of Conditional Configuration

Next, the sufficiency analysis of conditional configurations is to determine whether there is a combination of conditions as a sufficient condition for the occurrence of the result—that is, when the combination exists, the result must exist. This contains two sub-steps, truth table refinement and standard analysis. According to the results of necessity analysis, starting from condition combinations, we selected all the six variables as conditions and further analyzed different condition combinations that led to the occurrence of health-information-avoidance behavior. In this paper, the consistency threshold was set as 0.8, and the case frequency threshold was set as 1 [80]. 

After importing the data set into the fsqca3.0 software to construct the truth table, the complex solution, the intermediate solution, and the parsimonious solution were obtained. These complex solutions are based entirely on raw data, without using any logical remainders, and usually contain the largest number of configurations. The parsimonious solution uses all the logical remainders, and the result is the most concise. The intermediate solution is generally reported [81], because the intermediate solution does not follow the sample variables too strictly and also avoids overly concise violation cases, which can better explain the sample situation. 

We choose to report the intermediate solution as shown in Table 3, including three groups of configurations. According to Ragin and Fiss [81], “•” or “⚫” means that the condition exists, “⮾” or “⊗” means that the condition does not exist, and blank means that the condition is irrelevant to the result variable. By comparing the intermediate solution and the parsimonious solution, the core conditions and edge conditions in the antecedent conditions can be further determined. “⚫” and “⊗” represent the core condition, indicating that the presence/absence of the condition will have a greater impact on the outcome variable. “•” and “⮾” indicate edge conditions, indicating that the presence/absence of the condition has a weak effect on the outcome variable. The result of factor combinations in health-information-avoidance behavior is shown in Table 3.

As can be seen from Table 3, there are three configurations of antecedent conditions that can lead to avoid health information, and the overall consistency is about 0.82, which is higher than the acceptable level of 0.8.

### 4.3. Robustness Tests 

Robustness tests are a critical step in order to ensure the reliability of the results. A solution term is considered robust if the resulting solution term is similar to the original model under different operating options. In this paper, the method of adjusting the consistency threshold level was adopted to evaluate the robustness of the results [79]. The consistency level was increased by 0.05, and the frequency of cases remained unchanged. We found that the adjusted results still satisfied the overall consistency threshold. The overall coverage decreased slightly, and the number and contents of configurations had no obvious change. The analysis results had good robustness.

## 5. Discussion, Implications, and Limitations

This paper first clarified the concept, motivation, and tactics of health-information-avoidance behavior. After reviewing the literature, we summarized the factors related to avoidance and proposed to explore the role of influencing factors from a holistic perspective. Six variables, including health-information literacy, negative emotion, perceived information, health information presentation form, cross-platform distribution, and network information environment were considered. Then, fsQCA was used to analyze the health-information-avoidance cases obtained from the interviews, and the occurrence of avoidance was used as the outcome variable. From the analysis, we identified the influencing factors combined in a certain way leading to avoiding health information, and three configurations were obtained. 

### 5.1. Configuration M1: NE *~PI*HIP*~CPD*~NEQ

This configuration indicates that the combined factors for the occurrence of health-information-avoidance behavior are strong negative emotions, weak perception information, strong health-information presentation, less cross-platform distribution, and good network information environment. The results of this group show that, when an individual’s ability to perceive information is weak, they have less judgment on the quantity, quality, and content of information. 

In addition, users only use a single information channel and have a poor ability to judge and utilize information. Health information with a clear style of information presentation will clearly express a certain harm or benefit. In this case, the user cannot accept the content of the information, and the negative emotions are strong. In order to avoid such bad emotions or certain harmful consequences, the avoidance of health information happens. 

In our typical case, respondents indicated that, in the normal online information environment, although they knew the authenticity and validity of the content delivered by skin-disease-related health information, they would refuse to screen the pictures that were too real or highly popularized due to psychological discomfort. This message framing is at work. This is similar to the loss framework that indicates a terrible outcome if you have the skin diseases. It also shows that some purposes can be accomplished by changing the presentation of information. Some studies have applied framing effects [82] in the service of changing respondents’ COVID-19 vaccination intentions [83] or prompting healthy behaviors and beliefs with chronic disease [84].

### 5.2. Configuration M2: HIL *NE*HIP*~CPD*~NEQ

This configuration indicates that the combined factors for the occurrence of health-information-avoidance behavior are high health-information literacy, strong negative emotions, strong health-information-presentation form, less cross-platform distribution, and good network environment quality. A better network information environment provides users with the opportunity to obtain health information online; however, there are few information sources used, and individuals rely on their own health-information literacy to obtain, use, and evaluate health information. In this case, severe negative emotions can cause users to choose to avoid such health information when the information is presented in a more prominent form that shows serious illness or negative consequences. 

In our typical case, the presence of health-information literacy, negative emotions, and information representation is important. Those with lower health literacy as a burden were more likely to avoid information about COVID-19 [6]. This means that individual factors are important. The cognition and effects as the expression of how people think and feel result in the behavior. Usually, effect as a mediating variable influences the impact of cognition on behavior. More exploration from the perspective of cognitive psychology is also worth looking into. 

The core condition in configuration M1 and M2 is NE*HIP*~NEQ, negative emotions, health-information presentation, and a good network information environment. Correspondingly, ~PI and ~CPD in configuration M1 and HIL as well as ~CPD in configuration M2 are the edge conditions. This indicates that, in these two configurations, when users often use a single health information channel and fail to perceive that there are a large number of rumors and false health information on the internet, less-obvious information presentation forms and negative emotions can lead to avoiding health information. 

### 5.3. Configuration M3: HIL* NE*PI *CPD*NEQ

This configuration indicates that the combined factors for the occurrence of health-information-avoidance behavior are high health-information literacy, strong negative emotions, strong perceived information, a complex information environment, multi-distribution across platforms, and poor network environment quality. 

This configuration shows that the stronger the users’ ability to perceive information is, the more they can make use of their health-information literacy and rely on certain network search results to make their own judgments on information overload, reliability, authenticity, and relevance of information content, and they trust their own judgment or intuition. It is because of this trust that, when faced with negative information, discomfort is prominent, and this uncomfortable emotion will leave a psychological impact and make users eliminate this emotion by diverting attention, cheating, or something else. The core condition in this configuration is itself. 

In our typical cases, respondents believed they had some information literacy and frequently used multiple sources of information. However, the respondents remained skeptical of mobile we-media content—for instance, doubting the authenticity of online reviews [85]. While surfing the web, respondents selected videos that they thought were reliable; however, the surgical simulation video was considered too scary to watch again.

### 5.4. Implications

This study makes several key contributions. First, it shows that the occurrence of health-information-avoidance behavior is the result of a combination of internal and external factors. Individual cognition and negative emotions as internal factors will be affected by personal literacy, which is also an internal factor, while the presentation forms and characteristics of the health information and the network information environment are external factors that act together. 

Second, different from the linear relationship and net effects of causality in the traditional regression analysis method, this paper used qualitative comparative analysis to fully explore the causal complexity of health-information avoidance. The configuration analysis indicated that no one factor alone could trigger the avoidance of health information, and the same factors also presented different performance levels in different configuration types.

This study also offers implications for individuals and governments. Refusing to accept health information due to anxiety, fear, and other emotions or false health information, resulting in potential health risks, is an irrational behavior. In particular, for information-poor groups, such as farmers, the elderly, and people with lower education levels, their information needs are unclear, and they are less exposed to health information, resulting in an aggravation of the health digital divide. Therefore, as far as individuals are concerned, enhancing health information awareness, improving the ability to obtain, judge, and utilize health information, and reducing unnecessary health information anxiety and health-information avoidance can help people better respond to public health emergencies, improve self-health efficacy, and actively use health information for personal health management. 

### 5.5. Limitations

There are some limitations of this study. The interview samples are not sufficient, and most of them are about young groups. Some elderly groups who are susceptible to diseases and are easily affected by health information were not included in the scope of the study. Generalization of the results and conclusions needs to be cautious. User group types or general users are the research objects to obtain more combined analysis of primary and secondary data.

## 6. Conclusions

With the implementation of the “healthy China” strategy, health information channels have become increasingly diversified. Greater access to health information makes it easier to provide different types of information to meet health needs; however, a good network environment, high-quality information, and the matching of demand and supply of health information are challenges for the government. 

For example, under the normalization of the COVID-19 pandemic, people no longer pay great attention to the number of new infections and deaths; on the contrary, people under lockdown policies turn to mental health information. The government should strive to provide a good network information environment and high-quality health information, improve the capacity of cross-platform and multi-channel health information services, and promote the construction of the health-information service system. 

In the future, based on behavioral big data, it is worthwhile to attempt to use natural language processing, machine learning, and other methods to achieve more quantitative scoring of text data. Researchers are expected to design experiments to further explore the information framing effect with more types of participants to achieve a richer exploration of health information anxiety and avoidance behaviors.

## Figures and Tables

**Table 1:** The assignment criteria for all conditions.

Variables		Criteria	Value
Conditional Variables	Health Information Literacy (HIL)	Individuals with high health-information literacy.	1.00
		Individuals with certain health-information literacy.	0.67
		Individuals with basic health-information literacy.	0.33
		Individuals do not have health-information literacy.	0
	Negative Emotions (NE)	Individuals will have severe negative emotions when faced with health information, which will last for a long time.	1.00
		When faced with health information, individuals will have certain negative emotions, which will last for a short period of time.	0.67
		When faced with health information, individuals have mildly negative emotions, which will last for a short period of time.	0.33
		Individuals do not have negative emotions when faced with health information.	0
	Perceived Information (PI)	Individuals perceive information quantity, information quality and information content characteristics.	1.00
		Individuals have some perceptions about the quantity of information, the quality of information, and the characteristics of information content.	0.67
		Individuals are less aware of some aspects of the quantity of information, the quality of information and the characteristics of information content.	0.33
		Individuals have no perception of information quantity, information quality and information content characteristics.	0
	Heath-Information Presentation (HIP)	Individuals are very concerned about the presentation of health information.	1.00
		Individuals are more concerned about the presentation form of health information.	0.67
		Individuals generally care about the presentation of health information.	0.33
		Individuals do not care about the presentation of health information.	0
	Cross-platform Distribution (CPD)	Individuals frequently follow health information through three or more sources of information.	1.00
		Individuals often follow health information through one or two sources of information.	0.67
		Individuals pay less attention to health information through one or two sources of information.	0.33
		Individuals do not use information sources to follow health information.	0
	Network Environment Quality (NEQ)	There are a lot of health rumors or false health information on the internet.	1.00
		There are certain health rumors or false health information on the internet.	0.67
		There are fewer health rumors or false health information on the internet.	0.33
		There is no large number of health rumors or false health information on the internet.	0
Outcome Variable	Health-Information-Avoidance Behavior (HIAB)	Individuals achieve health-information avoidance.	1.00
		Individuals do not achieve health-information avoidance.	0

**Table 2:** The results of the single-factor necessity analysis.

Conditions	Consistency	Coverage
HIL	0.534000	0.687480
NE	0.541000	0.729848
PI	0.559250	0.744674
HIP	0.659000	0.806610
CPD	0.600250	0.720156
NEQ	0.600250	0.720156
~HIL	0.466000	0.778939
~NE	0.459000	0.724260
~PI	0.440750	0.706330
~HIP	0.341000	0.611111
~CPD	0.399750	0.738227
~NEQ	0.399750	0.738227

Notes: ‘~’ means “not”, non-set of conditions; case insensitivity in the fsQCA method.

**Table 3:** Health-information-avoidance behavior pathways.

Antecedent Conditions	Configurations		
	M1	M2	M3
HIL		•	•
NE	⚫	⚫	⚫
PI	⮾		⚫
HIP	⚫	⚫	⚫
CPD	⮾	⮾	•
NEQ	⊗	⊗	•
Consistency	0.829772	0.825265	0.800363
Raw coverage	0.2815	0.27275	0.33075
Unique coverage	0.0255	0.0085	0.08325
Overall solution consistency	0.822198		
Overall solution coverage	0.3815		

Notes: HIL denotes health-information literacy; NE denotes negative emotions; PI denotes perceived information; HIP denotes health-information presentation; CPD denotes cross-platform distribution; NEQ denotes network environment quality.

## Data Availability

All data generated or analyzed during this study are included in this manuscript.

## Abbreviations

QCA	Qualitative Comparative Analysis
fsQCA	fuzzy-set Qualitative Comparative Analysis
csQCA	crisp-set Qualitative Comparative Analysis
mvQCA	multi-value Qualitative Comparative Analysis
CIT	Critical Incident Technique
HIL	Health Information Literacy
NE	Negative Emotions
PI	Perceived Information
HIP	Heath-Information Presentation
CPD	Cross-platform Distribution
NEQ	Network Environment Quality
HIAB	Health-Information-Avoidance Behavior
